# Procalcitonin-guided antibiotic therapy in critically ill adults: a meta-analysis

**DOI:** 10.1186/s12879-017-2622-3

**Published:** 2017-07-24

**Authors:** Tao Zhang, Yan Wang, Qianting Yang, Yalin Dong

**Affiliations:** Department of Pharmacy, The First Affiliated Hospital of Xi’an Jiaotong University, Xi’an, 710061 China

**Keywords:** Procalcitonin, ICU, Meta-analysis, Trial Sequential Analysis

## Abstract

**Background:**

As a novel biomarker of inflammation, procalcitonin (PCT) has proven useful to guide antibiotic therapy in intensive care unit (ICU). However, there are controversial on mortality. The aim of this study was to evaluate the utility of PCT-guided antibiotic therapy in critically ill adults and determine whether studies are sufficient.

**Methods:**

A systematic search in PubMed, Embase and Cochrane was performed. We included only randomized controlled trials which compared the safety and efficacy between PCT-guided or standard antibiotic therapy groups in ICU adults. Trial sequential analysis and GARDE approach were performed.

**Results:**

Fifteen studies met our criteria for inclusion finally, with a cumulative number of 5486 ICU patients. There was no difference in 28-day mortality between two compared groups (*P* = 0.626), but significant decreases were observed in the duration of antibiotic therapy for the first episode of infection (*P* < 0.001) and length of hospitalization (*P* = 0.049). No significant deference was found in secondary endpoints except total duration of antibiotic therapy (*P* < 0.001). TSA revealed that the pooled sample sizes of 28-day mortality and the duration of antibiotic therapy for the first episode of infection exceeded the estimated required information size, but not the length of hospitalization.

**Conclusions:**

PCT-guided therapy is a better and safer algorithm to be applied into ICU patients, which appears no effect on 28-day mortality while performing preferable utility in reducing the duration of antibiotic therapy for the first episode of infection. More studies on these endpoints were not recommended.

**Electronic supplementary material:**

The online version of this article (doi:10.1186/s12879-017-2622-3) contains supplementary material, which is available to authorized users.

## Background

Based on statistics, more than 18 million patients of intensive care unit developed serious infections worldwide each year, which has been one of the major causes of death in ICU patients [1, 2]. However, there are some shortcomings with current gold standard for the diagnosis of infection - blood culture, such as low positive rate and time-consuming [3].

Procalcitonin (PCT) is a sort of inactive glycoprotein, composed by 116 amino acids [4, 5]. Compared with traditional indicators of bacterial infection, PCT emerges the superiority of sensitivity and specificity in diagnosis [6–8]. Many studies have demonstrated PCT was associated with type and severity of infections [9–14]. At first, PCT could help us to tell bacterial infection from others. Secondly, the level of PCT can be increased acutely by systemic infections, but not local bacterial infections or chronic nonspecific inflammation. So far, PCT has been applied in clinic in early diagnosis.

PCT-guided antibiotic therapy in ICU patients could decrease the total consumption and duration of antibiotic use without improvement in terms of safety such as mortality according to studies published previously [15, 16]. The latest meta-analysis about PCT-guided treatment in critical ill patients was conducted in 2011 [17], since then there have been extra new randomized controlled trials (RCTs). Recently, a newly published RCT with a large sample size showed mortality in PCT-guided arm was significantly lower than control arm, which aroused our interest if it could change the former conclusions [18]. Therefore, we conducted an update for the systematic review of PCT-guided antibiotic treatment, aiming at investigating the utility of PCT-based treatment algorithm in critically ill adults. Meanwhile, trial sequential analysis (TSA) were performed to detect the robustness of our results through calculating required information size.

## Methods

### Data source, search strategy and study election

Two viewers searched three English databases (PubMed, Embase, Cochrane) comprehensively from inception to 1 January 2017, using the search terms “procalcitonin”, “intensive care unit”, “randomized controlled trial” (see Additional file 1).

We included studies comparing the safety and efficacy between PCT-guided therapy and routine practice. We considered only RCTs reporting at least one of the following outcomes: 28-day mortality, duration of antibiotic therapy for the first episode of infection, length of hospitalization, mortality in hospitalization, total duration of antibiotic therapy, length of ICU stay and recurrences, among which the first three and the latter four are primary and second endpoints respectively. We did not include studies about neonate patients, due to its considerable differences in diagnosis and therapy of compared to adults. In addition, studies in pediatrics and emergency departments were also excluded.

Literature search and evaluation of potential eligible articles were conducted by two investigators independently with no language restrictions. Any disagreement regarding study eligibility was resolved via discussion by two reviewers or consultation with a senior researcher when necessary.

### Data extraction and quality assessment

Two researchers extracted all of the data using a standardized collection process developed in Microsoft Excel 2013. Basic characteristics and outcome data of RCTs were extracted, including the following items: the first author, year of publication, country, study design, ICU type, inclusion criteria, PCT measurement method, sample size, details of intervention and control, severity of illness scores on study enrollment and outcomes mentioned above. The corresponding authors were contacted by E-mail if important data were unavailable.

Reviewers independently screened all full text articles retrieved to assess risk of bias following the recommendations in the Cochrane handbook for systematic reviews of interventions, which includes seven domains: random sequence generation, allocation concealment, blinding of participants and personnel, blinding of outcome assessment, incomplete outcome data, selective outcome reporting, and other sources of bias. The results of assessment were categorized as “low risk of bias,” “high risk of biases,” or “unclear risk of bias.” The overall quality of evidence for each outcome was assessed further at the outcome level using the Grades of Recommendation, Assessment, Development, and Evaluation (GRADE) approach. Any disagreement was resolved by discussion.

### Statistical synthesis and analysis

The STATA software (STATA/MP 13.0) was used for meta-analysis and forest plots was applied for display of results. In order to ensure the reliability of the results, we conducted analysis only for the same outcome reported in more than two RCTs. The chi-squared tests and I^2^ inconsistency statistics were used for between-trial heterogeneity tests. A *P*-value of <0.10 was thought to indicate significant heterogeneity, whereas I^2^ values of 0–24.9%, 25–49.9%, 50–74%, and 75–100% were considered to represent none, low, moderate, and severe statistical inconsistency, respectively. Characteristics of included studies were summarized using frequencies and percentages for binary variables, as well as means and standard deviations (SDs) for continuous variables which we would imputed if the study provided only medians and interquartile ranges. Continuous outcomes were analyzed using weighted mean differences (WMD) and 95% confidence intervals (CIs). For all analyses performed, a random effect model (REM) was used to calculate pooled odds ratios (ORs), 95% CIs, and values regardless of the heterogeneity [19]. Statistical significance was set at a two-sided *P* < 0.05. Clinical heterogeneity between trials was addressed by further meta-regression which focused on severity of illness scores on study enrollment, sample size and the proportion of sepsis patients. In addition, sensitivity analyses for the primary outcome were carried out to evaluate the consistency of the results by omitting one study at a time. A funnel plot method combined with the Begg’s text and Egger’s test for asymmetry was also used to assess the potential of publication bias if the number of studies was enough to do so (*n* ≥ 10).

### Trial sequential analysis

To avoid increasing type I error resulting from repetitive significance test of sparse and accumulated data, we also conducted trial sequential analysis for primary outcomes which adjusted for random error risk, to calculate the required number of participants (required information size, RIS) and the cumulative Z-curve’s eventual breach of relevant trial sequential monitoring boundaries, using TSA v0.9.5.5. The RIS of the trial sequential analysis was based on 5% risk of a type I error and 20% risk of a type II error (power of 80%). Statistical significance was set at a two-sided *P* < 0.05.

## Results

### Description of included studies

#### Search results

**Fig. 1:**
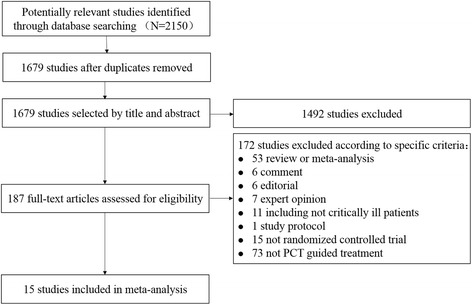
Flow chart of the identification of eligible trial

A total of 2150 records were identified in literature databases according to the search strategy (375, 1614, 161 in PubMed, Embase, Cochrane respectively). After excluding 471 duplicates, and 1492 irrelevances by reviewing the titles and abstracts of articles, we reviewed 187 full text articles retrieved which fulfilled the eligibility criteria, and 15 articles were included in this review eventually [18, 20–33]. The details of search strategy and screening process are shown in Additional file 1 and Fig. 1 respectively.

#### Study characteristics

General characteristics are displayed in Table 1 below. Fifteen RCTs studies from different countries were included with a total number of 5486 critically ill adult patients, among which 2748 and 2738 participants were enrolled in PCT-guided and standard arm respectively. All the include studies were published between 2007 and 2016, with 7 single-center studies and 8 multi-center studies. The sample sizes of each study ranged from 27 to 1546. Overall, studies reported 75.4% of patients with sepsis; ranged from 0 to 100%. The language used in the all eligible studies is in English but one is in Chinese [23]. Fourteen studies provided at least one of following scores to describe the severity of illness on study enrollment: Acute Physiology and Chronic Health Evaluation II (APACHE II), Acute Physiology and Chronic Health Evaluation IV (APACHE IV), Simplified Acute Physiology Score II (SAPS II), Simplified Acute Physiology Score III (SAPS III), Sequential Organ Failure Assessment (SOFA). In thirteen RCTs (86.7%), the attending physician were allowed to overrule the algorithm, which means they were free to decide whether to continue antibiotic treatment or not in patients of control group who had reached thresholds. PCT serum level was measured using immunochromatographic technique (PCT-Q, Brahms) in only one study, automated immunoassay (VIDAS PCT, Brahms) in two studies, Luminescence immunoassay (PCT LIA, Brahms) in three studies and time-resolved amplified cryptate emission technology (Kryptor PCT, Brahms) in eight studies and. Especially, all methods mentioned above are used in another study except the Immunochromatographic technique. Ten of the fifteen studies reported a priori power calculation with targeted sample size achieved for the primary outcome of interest while the remaining articles did not report any information about a power calculation. Additional characters of included RCTs see Additional file 2.

**Table 1:** Main characteristics of the included RCTs: PCT arms versus control arms

Fist Author (Year)	Country	Number of Patients	Study Design/ Setting	PCT Measurement	Overrule the Algorithm or Not	Severity of Illness on Study Enrollment^e^
Svoboda [33] (2007)	Czech Republic	38/34	RCT, single-center/ 4 surgical ICUs	Immunochromatographic technique^a^	Yes	APACHE II: 15.7 (7.9) /17.3 (9.3)
Nobre [32] (2008)	Swizerland	39/40	RCT, single-center/ 1 mixed medical-surgical ICU	Time-resolved amplified cryptate emission^b^	Yes	SOFA: 6.4 (3.3) /6.6 (3.0) SAPS III: 68.5 (12.1) /70.1 (13.1)
Hochreiter [31] (2009)	Germany	57/53	RCT, single-center/ 1 surgical ICU	Luminescence immunoassay^c^	Yes	APACHE II: 40.1 (17.1) /40.5 (15.1) SAPS II: 40.1 (17.1) /40.5 (15.1)
Schroeder [30] (2009)	Germany	14/13	RCT, single-center/ 1 surgical ICU	Luminescence immunoassay^c^	Yes	SAPS II: 45.6 (18.5) /53.7 (14.7)
Stolz [29] (2009)	USA	51/50	RCT, multi-center/ 7 mixed medical-surgical ICUs	Time-resolved amplified cryptate emission^b^	Yes	SOFA: 7.3 (3.4) /8.2 (3.4) SAPS II: 42 (13) /45 (14)
Bouadma [28] (2010)	France	307/314	RCT, multi-center/ 7 ICUs (5 medical, 2 surgical)	Time-resolved amplified cryptate emission^b^	Yes	SOFA: 7.5 (4.4) /7.2 (4.4) SAPS II: 43.8 (16.1) /43.4 (15.4)
Layios [26] (2012)	Belgium	258/251	RCT, single-center/ 5 ICUs	Time-resolved amplified cryptate emission^b^	Yes	APACHE II: 39.3 (16.3) /39 (16.7)
Jensen [27] (2011)	Denmark	604/596	RCT, multi-center/ 9 mixed medical-surgical ICUs	Time-resolved amplified cryptate emission^b^	Yes	NA
Liu [23] (2013)	China	42/40	RCT, single-center/ 1 mixed medical-surgical ICU	Luminescence immunoassay ^c^	No	APACHE II: 21.6 (4.3) /18.5 (3.6)
Annane [25] (2013)	France	30/28	RCT, multi-center/ 8 mixed medical-surgical ICUs	Time-resolved amplified cryptate emission^b^	No (at 5 days after antibiotic initiated)	SOFA: 9.5 (8.5–11.0) /10 (8–11)^f^ SAPS II: 32.5 (27–47) /43 (32–52)^f^
Deliberato [24] (2013)	Brazil	42/39	RCT, single-center/ 1 mixed medical-surgical ICU	Automated immunoassay^d^	Yes	SOFA: 6.29 (2.85) /5.38 (3.33) SAPA II: 56.9 (11.68) /53.77 (12.33)
Shehabi [22] (2014)	Australia	196/198	RCT, multi-center/ 11 mixed medical-surgical ICUs	Automated immunoassay^d^	Yes	APACHE II: 21.2 (7.8) /20.9 (7.1) SOFA: 6.0 (3.0–9.0) /6.0 (3.0–8.0)^f^
Najafi [21] (2014)	Iran	30/30	RCT, single-center/ 1 mixed medical-surgical ICU	Time-resolved amplified cryptate emission^b^	Yes	APACHE II: 11.9 (9.3) /13.3 (7.9) SOFA: 5.4 (3.6) /5.7 (2.8)
Jong [18] (2016)	Netherlands	761/785	RCT, multi-center/ 15 mixed medical-surgical ICUs	Time-resolved amplified cryptate emission^b^ or Luminescence immunoassay^c^ or Automated Immunoassay^d^	Yes	APACHE IV: 72.0 (52.0–92.0) /71.0 (55.0–95.0)^f^ SOFA: 6.0 (3.0–9.0) /6.0 (4.0–9.0)^f^
Bloos [20] (2016)	Germany	279/267	RCT, multi-center/ 33 mixed medical-surgical ICUs	Time-resolved amplified cryptate emission^b^	Yes	APACHE II: 24.2 (7.2) /24.4 (7.7) SOFA: 10.0 (3.3) /9.9 (3.3)

^a^PCT- Q, Brahms

^b^Kryptor PCT, Brahms

^c^PCT LIA, Brahms

^d^VIDAS PCT, Brahms

^e^Mean (SD)

^f^median (interquartile range)

#### Assessment of risk of bias

**Fig. 2:**
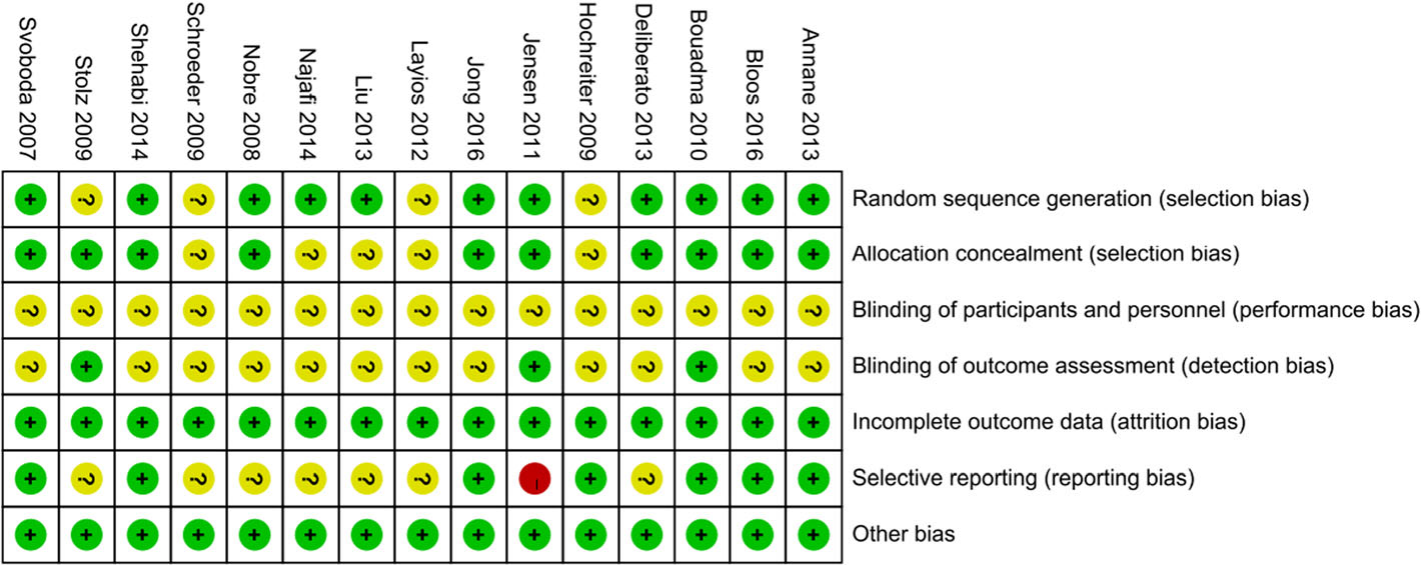
The risk of bias summary or review of judgments on each risk of bias item for each included study. (+, low risk of bias; −, high risk of bias;?, unclear risk of bias)

Figure 2 details the risk of bias assessment for the included studies. In brief, the overall risk of bias was moderate according to the Cochrane Collaboration tool, but one study showed a high risk of bias due to selective reporting. We conducted assessment of publication bias only for 28-day mortality, length of hospitalization and length of ICU stay because of the low number of included studies for other endpoints. No significant publication bias was identified in the visual funnel plot (see Additional file 3). These observations were confirmed in both of Begg’s test and Egger’s test (Table 3).

### Effects of the interventions

#### Primary outcomes

##### 28-day mortality

**Fig. 3:**
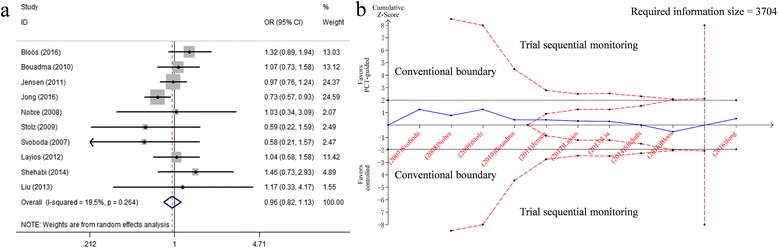
Effects of PCT-guided antibiotics therapy on ICU patients for the 28-day mortality. **a** Forest plots; **b** Trial sequential analysis

Ten studies assessed the 28-day mortality and no difference was found between the compared groups (*n* = 5155, OR 0.96, 95%CI 0.82 to 1.13, *P* = 0.626, I^2^ = 19.5%, *P* = 0.264; low quality) (Fig. 3a, Tables 2 and 3). However, it is noteworthy that the result of one study with the largest sample size showed a significant decrease in PCT-guided group.

**Table 2:** Primary and secondary outcomes of the included trials: PCT arms versus control arms

Study (year)	Primary endpoints			Second endpoints			
	28-day mortality	duration of antibiotic therap by for the first episode of infection^a^	length of hospitalization^a^	mortality in hospitalization	total duration of antibiotic therapy ^a^	length of ICU stay ^a^	Recurrences
Svoboda (2007) [33]	10/13	NA	NA	NA	NA	16.1 (6.9) /19.4 (8.9)	NA
Nobre (2008) [32]	8/8	6.0 (7.8) /9.5 (7.8)	17.0 (23.3) /23.5 (9.8)	9/9/	8.0 (5.8) /14.0 (8.3)	4.0 (5.0) /7.0 (22.5)	1/1/
Hochreiter (2009) [31]	NA	5.9 (1.7) /7.9 (0.5)	NA	15/14	5.9 (1.7) /7.9 (0.5)	15.5 (12.5) /17.7 (10.1)	NA
Schroeder (2009) [30]	NA	6.6 (1.1) /8.3 (0.7)	NA	3/3	6.6 (1.1) /8.3 (0.8)	16.4 (8.3) /16.7 (5.6)	NA
Stolz (2009) [29]	8/12	3.0 (2.0) /5.0 (4.1)	26.0 (10.4) /26.0 (4.1)	10/14	10.0 (2.5) /15.0 (3.3)	NA	6/11
Bouadma (2010) [28]	65/64	6.1 (6.0) /9.9 (7.1)	26.1 (19.3) /26.4 (18.3)	NA	10.3 (7.7) /13.3 (7.6)	15.9 (16.1) /14.4 (4.1)	20/16
Layios (2012) [26]	56/53	NA	NA	NA	NA	7.0 (8.9) /7.0 (10.4)	NA
Jensen (2011) [27]	190/191	NA	NA	NA	NA	6.0 (6.7) /5.0 (5.9)	NA
Liu (2013) [23]	6/5	8.1 (0.3) /9.3 (0.3)	27.0 (4.9) /32.0 (5.4)	NA	8.1 (0.3) /9.3 (0.3)	12.0 (2.9) /14.0 (2.7)	3/1
Annane (2013) [25]	NA	NA	27.0 (29.6) /33.0 (43.0)	7/10	NA	22.0 (25.2) /23.0 (37.0)	NA
Deliberato (2013) [24]	NA	NA	11.0 (11) /11.0 (56.5)	2/4	NA	3.5 (14.0) /3.0 (6.8)	2/1
Shehabi (2014) [22]	21/15	NA	15.0 (14.8) /17.0 (16.3)	30/26	NA	6.0 (4.8) /6.0 (4.4)	6/12
Najafi (2015) [21]	NA	NA	20.0 (9.0) /22.0 (14.8)	5/4	NA	4.0 (4.5) /6.0 (6.5)	NA
Jong (2016) [18]	149/196	NA	22.0 (19.3) /22.0 (20.7)	NA	NA	8.5 (8.9) /9.0 (9.6)	38/23
Bloos (2016) [20]	77/60	7.0 (6.7) /7.0 (7.4)	29.0 (23.7) /29.0 (24.4)	NA	NA	12.0 (14.1) /12.0 (11.1)	NA

^a^Mean (SD); NA, not available

**Table 3:** Meta-analysis of aggregate data

Outcome	Studies	Patients	Effect size OR (CI)	*P* value	Heterogeneity		Publication bias (*P* value)		Quality of evidences
					I^2^ (%)	*P* value	Begg’s test	Egger’s test	
Primary endpoints									
28-day mortality	10	5155	0.96 (0.82, 1.13)	0.626	19.5	0.264	0.929	0.534	low
Duration of antibiotic therapy for the first episode of infection	7	1566	-1.83 (−2.51, −1,15)	< 0.001	85.8	0.000	-	-	moderate
Length of hospitalization	10	3571	-1.61 (−3.20, 0.01)	0.049	42.2	0.077	0.180	0.778	moderate
Second endpoints									
Mortality in hospitalization	8	913	0.94 (0.66, 1.32)	0.815	0.0	0.970	-	-	low
Total duration of antibiotic therapy	6	1020	-2.68 (−3.36, −1.73)	< 0.001	92.8	< 0.001	-	-	moderate
Length of ICU stay	14	5385	−0.33 (−1.09, 0.42)	0.384	54.1	0.008	0.412	0.178	moderate
Recurrences	7	2867	1.11 (0.69, 1.79)	0.676	28.3	0.212	-	-	moderate

*OR* odds ratio, *CI* confidence interval

TSA showed that though the Z-curve did not cross the conventional boundary, the cumulative crossed the trial sequential monitoring boundary, and the pooled sample size of patients exceeded the estimated RIS (Z < 1.96, *P* > 0.05) (Fig. 3b), confirming the results of the meta-analysis that intervention group lacks of the effect to reduce 28-day mortality.

##### Duration of antibiotic therapy for the first episode of infection

**Fig. 4:**
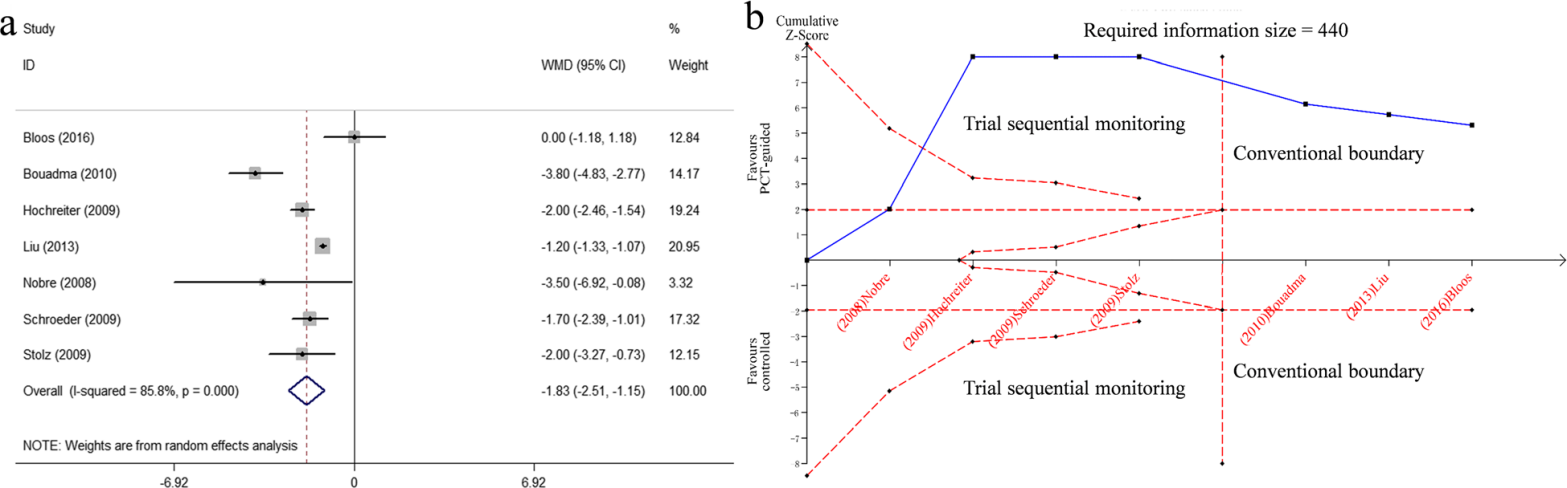
Effects of PCT-guided antibiotics therapy on ICU patients for the antibiotic duration for the first episode of infection. **a** Forest plots; **b** Trial sequential analysis

Data regarding the duration of antibiotic therapy for the first episode of infection were reported in seven of the fifteen included studies. An approximate 2-day shorter was observed in patients assigned to the PCT-guided group (*n* = 1566, WMD -1.83, 95%CI 2.51 to −1.15, *P* < 0.001; moderate quality) (Fig. 4a, Tables 2 and 3).

In the TSA, the calculated RIS was 440, exceeded by accrued sample size. Meanwhile, the cumulative Z-curve crossed both the conventional boundary and trial sequential monitoring boundary (Z > 1.96, *P* < 0.05), indicating that the evidence was reliable and conclusive, and no more study was necessary (Fig. 4b).

##### Length of hospitalization

**Fig. 5:**
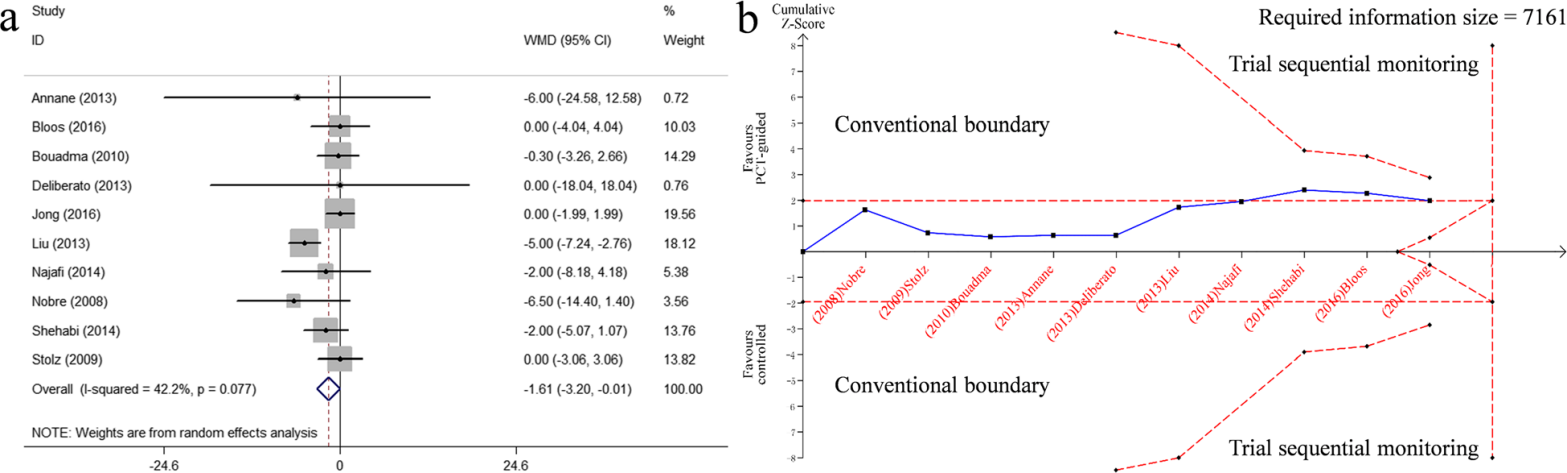
Effects of PCT-guided antibiotics therapy on ICU patients for the length of hospitalization. **a** Forest plots; **b** Trial sequential analysis

Data on the length of hospitalization was assessed in ten RCTs. We found a weak but significant difference between the compared arms. The length of hospitalization in the PCT-guided groups was reduced by 1.6 days compared to that in the standard cares with low heterogeneity (*n* = 3571, WMD -1.61, 95%CI -3.20 to −0.01, *P* = 0.049; I^2^ = 42.2%, *P* = 0.077; moderate quality) (Fig. 5a, Tables 2 and 3).

The result of TSA showed the estimated RIS was far more than pooled sample size of participants (7161 VS. 3571). Moreover, the cumulative Z-curve crossed only the conventional boundary a bit, suggesting the result of the meta-analysis was potentially spurious evidence of effect (Z > 1.96, *P* < 0.05) (Fig. 5b). Further RCTs are required and the optimal sample size needed to detect a plausible treatment effect.

#### Secondary outcomes

Compared with standard-care group, there was a significant decrease in the total duration of antibiotic therapy in PCT-guided therapy (*n* = 1020, OR -2.68, 95%CI -3.36 to −1.73, *P* < 0.001) (Table 3), whereas the other secondary outcomes were non-significant between two compared groups (see Additional file 4). The quality of evidence for each of the secondary outcome was moderate except mortality in hospitalization. The overall risk of bias of quality of evidences rated by the GRADE approach were summarized in Table 3.

#### Heterogeneity and Sensitivity analysis

The heterogeneity analysis focusing on severity of illness scores on study enrollment, sample size and the proportion of sepsis patients could not address the heterogeneity of meta-analysis for the primary outcomes, though the duration of antibiotic therapy for the first episode of infection and length of hospitalization presented severe (I^2^ = 85.8%) and low (I^2^ = 42.2%) statistical heterogeneity respectively. The results are shown in Table 3.

By removing each individual trial from the meta-analysis in sensitivity analyses, the conclusion of 28-day mortality and duration of antibiotic therapy for the first episode of infection were not affected by the exclusion of any trial. However, there were six of ten studies of length of hospitalization in which any trial excluded could cause result reversal (see Additional file 5).

## Discussion

Our principal findings were less duration of antibiotic therapy for the first episode of infection, length of hospitalization and total duration of antibiotic therapy in PCT-guided group with no improvement in mortality. More studies conducted to investigate the efficacy of PCT-guided treatment on 28-day mortality and duration of antibiotic therapy for the first episode of infection is not necessary, because adequate sample size had been achieved on these two outcomes. The results further encouraged clinicians to make therapeutic regimen according to the level of PCT, but not only decided by clinical experience.

To our knowledge, this is the first comprehensive meta-analysis combined with TSA to compare the utility of PCT-guided treatment and standard treatment in critically ill adults. In our study, we analyzed distinct outcomes separately without substitution in order to increase endpoint similarities. Moreover, the sample size had multiplied four times compared with a former meta-analysis [16], making our results convincing. After including more studies, we found PCT-guided treatment could reduce duration of antibiotic therapy for the first episode of infection, which was in accordance with other studies [15, 16], and length of hospitalization significantly. In addition, conducting TSA to test whether the sufficient information size had been reached is a highlight of our study, minimizing potentially false positive results and providing the basis for the further studies.

All studies published before Jong’s research showed no improvement in 28-day mortality using PCT-guided treatment [21–33]. Although Jong’s [18], a recent study with the biggest sample size, showed significant decrease in 28-day mortality (*P* = 0.0122), making us more confident in the safety of PCT-guided treatment, we still failed to found any significant difference through a comprehensive analysis. The possible reason was clinicians made adequate judges on the types and causes of infection, which reduced the risk of antibiotics misuse in Jong’s. Hence, that ICU patients were prevented from being suffered from the toxic effect that unnecessary antibiotics brought might have contributed to the survival benefit. TSA revealed that a stable conclusion had been made on 28-day mortality before 2011 and the later studies just confirmed it further. With the development of medical technology rapidly, the duration of antibiotic therapy is probably shorter nowadays, especially during a first infectious episode. Our study showed PCT-guided antibiotics therapy could further reduce duration of antibiotic therapy for the first episode of infection and total duration of antibiotic therapy. Interestingly, Jensen et al. showed an increased use of antibiotics by taking PCT-guided antimicrobial escalation in PCT group [27]. It is noting that other trials neither adopted this strategy nor draw similar conclusion [18, 22, 26, 32], suggesting that using the strategy of increasing the antimicrobial spectrum of antibiotics as an intervention in ICU patients may not be appropriate. What deserved to be noticed was that length of hospitalization showed decrease in PCT-guided therapy with statistical significance, but only one of the ten study obtained positive result [23], while the remains are negative. The findings of heterogeneity analyses including SOFA score and the proportion of sepsis patients did not change this association. Nevertheless, by synthesizing data from studies with big sample size (*n* > 100), we found no difference in length of hospitalization between PCT-guided treatment and standard practice (see Additional file 6), which may be the result from that study with small sample size was more likely to be effected by random error. Moreover, there were six of ten studies of length of hospitalization among which removing any individual trial excluded would cause result reversal, and five have small sample size (*n* < 100, 21, 23–25, 32]. Even if the remain one has sample size of 394, it is still considered small [22]. Thus, whether PCT-guided algorithm really could reduce length of hospitalization is still to be determined. TSA indicated the cumulative Z-curve of the length of hospitalization just crossed the conventional boundary but not the trial sequential monitoring boundary, which suggested more studies are needed on this endpoint.

It was reported that the dynamic changes of PCT level are more valuable than the absolute on prognosis of sepsis [34, 35]. What’s more, PCT-guided treatment on sepsis had been listed into the Diagnostic and Therapeutic Guidance in several developed countries [36, 37]. However, PCT level might also be elevated by other reasons except bacterial infections, including operation, trauma, burns, cardiac shock, multiple organs dysfunction syndrome, pancreatitis, autoimmune diseases, etc. [38–41]. The type of pathogens should also be put into consideration when low PCT level of patients were obtained. The reason why different pathogens cause differences in PCT levels might result from different interactions between the pathogens themselves and the host cells [42]. Actually, an ideal biomarker in the infectious diseases would have all characters including diagnostic, prognostic, follow-up of therapy, and is easily and rapidly available from clinic [43]. PCT could be considered as the one approximately. On the one hand, according to existing researches, we suggest clinicians should identify whether patients are infected by bacteria by making use of PCT, which is especially important for ICU patients in the early stages. On the other hand, clinicians should combine the level and change of PCT with other classic markers of inflammatory syndrome to make individual scheme of treatment, which is very meaningful for controlling infection in clinical practice.

We noticed there has been many studies about cost-effectiveness of PCT [44, 45], but they merely focused on various fees and did not use adequate methodology to evaluate whether PCT-guided antibiotic therapy could lead to emergence of multiresistant strains, which is very necessary and important for confirming the place of PCT in clinical practice. Therefore, we hope more researches in this aspect would be conducted in the future and it is best to take into account the country effect.

We also considered some limitations of this study. These include: 1) In the randomized studies, the standard therapy group is quite poorly defined in the different papers and PCT-guided treatment algorithms were not only variable, but also allowed to be overruled by physicians in most studies, which made the clinical treatment program much more flexible, increasing between-trial heterogeneity. 2) Relatively speaking, we still included a limited number of studies. Because the purpose of each study is different, not all the literatures reported all the endpoints we need. For instance, the total duration of antibiotic therapy, one of the three primary endpoints, was provided in only six studies. Interestingly, we still obtained a confirmed result. 3) Some data of outcomes could not be obtained directly. For this reason, we imputed means and SDs through appropriate statistical method. 4) The determination methods of PCT level were different. The patients whose values of PCT level are around the thresholds are more likely to accept different treatment regimens even if the PCT-guided treatment algorithms are consistent. We expect later researches to uniform standard of measurement methods so as to reduce the bias of outcomes. 5) The RCTs cannot perform blinding to clinicians due to inherent characteristics. Hence, we do cannot exclude the possibility that clinicians tended to manage more rigorously in the PCT group. However, PCT-based algorithm should be responsible for the effect on the duration of antibiotic use in a great degree, because clinicians were not allowed to overrule the algorithm unless they have well-founded explanation of their decisions.

## Conclusion

In conclusion, PCT-guided treatment could reduce the duration of antibiotic therapy for the first episode of infection in adult critically ill patients, and shows non-inferiority in mortality compared with standard practice, which is important to reduce the drug-related side effects and escalating costs. Sufficient sample size had been achieved and more investigations are not recommended on 28-day mortality and the duration of antibiotic therapy for the first episode of infection.

## Additional files

Additional file 1: Search strategy of electronic databases. (DOCX 13 kb)

Additional file 2: Inclusion criteria and treatment strategy in included studies. (DOCX 17 kb)

Additional file 3: Funnel plots for outcomes included more than 10 studies. (a) 28-day mortality; (b) length of hospitalization; (c) length of ICU stay. (TIFF 486 kb)

Additional file 4: Forest plots of second outcomes. (a) mortality in hospitalization; (b) total duration of antibiotic therapy; (c) length of ICU stay; (d) recurrences. (TIFF 1362 kb)

Additional file 5: Sensitivity analysis for primary outcomes by removing each trail. (a) 28-day mortality; (b) duration of antibiotic therapy for the first episode of infection; (c) length of hospitalization. (TIFF 288 kb)

Additional file 6: Subgroup analysis for length of hospitalization: sample size >100 and sample size <100. (TIFF 511 kb)

## Abbreviations

CI: Confidence interval
GRADE: Grades of recommendation, assessment, development, and evaluation
Hrs: Hours
ICU: Intensive care unit
OR: Odds ratio
PCT: Procalcitonin
RCT: Randomized controlled trial
REM: Random effect model
SD: Standard deviations
TSA: Trial sequential analysis
WMD: Weighted mean differences
Yrs: Years
