# Correction to: Procalcitonin-guided antibiotic therapy in critically ill adults: a meta-analysis

**DOI:** 10.1186/s12879-019-4707-7

**Published:** 2019-12-23

**Authors:** Tao Zhang, Yan Wang, Qianting Yang, Yalin Dong

**Affiliations:** Department of Pharmacy, The First Affiliated Hospital of Xi’an Jiaotong University, Xi’an, 710061 China

**Correction to: BMC Infect Dis**

**https://doi.org/10.1186/s12879-017-2622-3**

After publication of the original article [1], the authors have reported that the software developers had used a new amended version of the software called “v0.9.5.10” instead of “v0.9.5.5”, which is referred to in the original article.

Thus, the authors have conducted trial sequential analysis with new version of the software for the two continuous end-points. All corrections are following:

1. “v0.9.5.5” in the Trial sequential analysis (methods section) is corrected to “v0.9.5.10”.

2. The estimated required information size of Length of hospitalization (7161, results section) is corrected to “7242”.

3. Fig. 5 is below updated.

**Fig. 5:**
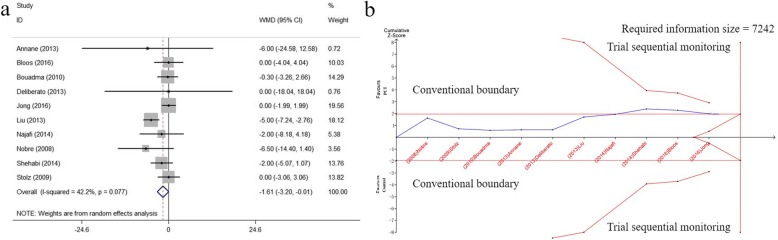
Effects of PCT-guided antibiotics therapy on ICU patients for the length of hospitalization. **a** Forest plots; **b** Trial sequential analysis

The conclusions are as same as published in the original article.

[1] References: Zhang et al. BMC Infectious Diseases (2017) 17:514, DOI: 10.1186/s12879-017-2622-3
