# Supplementary material for: Procalcitonin-guided antibiotic therapy in critically ill adults: a meta-analysis
Source: BMC Infect Dis. 2017 Jul 24;17:514. doi: 10.1186/s12879-017-2622-3 (PMC5525369; doi:10.1186/s12879-017-2622-3)
Supplement: Supplementary file 1 — Search strategy of electronic databases. (DOCX 13 kb) [file 12879_2017_2622_MOESM1_ESM.docx]

**Search strategy of electronic databases (PubMed as sample):**

**#1** Critical Care [Mesh] OR Critical Care Nursing [Mesh] OR Critical Care Outcomes [Mesh]

**#2** critic* [Text Word]

**#3** intensive [Text Word]

**#4** surgic* [Text Word]

**#5** #1 or #2 or #3 or #4

**#6** procalcitonin [Supplementary Concept]

**#7** procalcitonin [Text Word]

**#8** #6 or #7

**#9** Randomized Controlled Trial [Publication Type] OR Randomized Controlled Trials as Topic [Mesh] OR Controlled Clinical Trial [Publication Type]

**#10** random* [Text Word]

**#11** control* [Text Word]

**#12** trial [Text Word]

**#13** #9 or #10 or #11 or#12

**#14** #5 and #18 and #13
