# Supplementary material for: Procalcitonin-guided antibiotic therapy in critically ill adults: a meta-analysis
Source: BMC Infect Dis. 2017 Jul 24;17:514. doi: 10.1186/s12879-017-2622-3 (PMC5525369; doi:10.1186/s12879-017-2622-3)
Supplement: Supplementary file 2 — Inclusion criteria and treatment strategy in included studies. (DOCX 17 kb) [file 12879_2017_2622_MOESM2_ESM.docx]

**Table S1:** Inclusion criteria and treatment strategy in included studies

| **Fist Author**  **(Year)** | **Inclusion Criteria** | **Strategy in PCT Arm** | **Strategy in Control Arm** |
| --- | --- | --- | --- |
| Svoboda  (2007) | 1) > 18 yrs; 2) patients with severe sepsis after multiple trauma or abdominal surgery | 1) PCT > 2 μg/L: change of antibiotics and catheters ; 2) PCT < 2 μg/L: ultrasonography and/or CT followed by repeated surgical treatment if localized infection was confirmed | Treatment according to the standard protocol of the hospital |
| Nobre  (2008) | all patients admitted to ICU with suspected severe sepsis or septic shock | **Antibiotic discontinuation:** 1) PCT dropped by > 90% compared with the baseline peak level or PCT level < 0.25 μg/L reevaluated at Day 5 if baseline ≥ 1 μg/L; 2) PCT < 0.1 μg/L reevaluated at Day 3 if PCT baseline < 1 μg/L | Regimens according to empirical rules |
| Hochreiter  (2009) | 1) infections caused by confirmed or highly suspected bacterial; 2) patients meeting ≥ 2 SIRS criteria | **Antibiotic discontinuation:** 1) clinical signs and symptoms of infection improved and PCT < 1 μg/L; 2) PCT > 1 μg/L and dropped 25% to 35% of the baseline within three days | Antibiotic treatment according to standard regimen over 8 days |
| Schroeder  (2009) | 1) patients admitted to ICU with severe sepsis after abdominal surgery; 2) patients receiving antibiotic treatment | **Antibiotic discontinuation:** clinical signs of infection improved and the PCT <1 μg/L or dropped to < 35% of the baseline within three days | Regimens according to empirical rules |
| Stolz  (2009) | 1) ICU patients receiving mechanical ventilation for ≥ 48 hrs; 2) > 18 yrs; 3) clinically diagnosed VAP | **Antibiotic discontinuation:** PCT < 0.25 μg/L;  **Antibiotic reduction or discontinuation:** PCT between 0.25 and 0.5 μg/L or dropped by > 80% of the baseline;  **Antibiotic continuation:** PCT > 0.5 μg/L or dropped by < 80% of the baseline | Regimens according to empirical rules |
| Bouadma  (2010) | 1) all adults admitted to ICU with suspected bacterial infections or sepsis; 2) no antibiotic treatment before inclusion or < 24 hrs and the interval between admission and inclusion < 12 hrs | **Antibiotic discontinuation:** PCT dropped by > 80% of the peak value or absolute value < 0.5 μg/L | Antibiotic treatment according to international  and local guidelines |
| Layios  (2012) | 1) > 18 yrs; 2) hospitalized for > 2 days in ICU | **More strongly discouraged:** PCT < 0.25 µg/L  **Less strongly discouraged:** PCT < 0.50 µg/L  **More recommended:** PCT > 1 µg/L  **Less recommended:** PCT > 0.50 µg/L | antibiotic treatment according to standard regimen |
| Jensen  (2011) | 1) ≥ 18 yrs; 2) enrollment within 24 hrs after ICU admission; 3) expected ICU stay ≥ 24 hrs | When “alert procalcitonin”^a^ occurred:  1) substantially increasing the antimicrobial spectrum covered; 2) intensifying the diagnostic effort to find uncontrolled sources of infection | Antimicrobial treatment according to clinical guidelines |
| Liu  (2013) | 1) ≥ 18 yrs; 2) suspected sepsis; 3) informed consent obtained | **Antibiotic discontinuation:** 1) PCT dropped by ≥ 90% compared with the baseline; 2) PCT < 0.25 μg/L | Antibiotic treatment according to the guidelines of treating sepsis |
| Annane  (2013) | all consecutive adults admitted to ICUs within 48 hrs meeting one of following criteria: 1) systemic inflammatory response syndrome; 2) acute dysfunction of at least one organ; 3) absence of indisputable clinical infection; 4) negative microbial cultures | Medical patients *(Surgical patients)*  **Antibiotic discontinuation:** PCT < 0.25 μg/L *(4 μg/L)*  **Antibiotic strongly discouraged:** PCT of 0.25-0.5 μg/L  **Antibiotic recommended:** PCT of 0.5-5 μg/L *(4-9μg/L)*  **Antibiotic strongly recommended:** PCT ≥ 5 μg/L *(9 μg/L)* | Antibiotic treatment according to attending physician |
| Deliberato  (2013) | Patients confirmed infections with sepsis, severe sepsis or septic shock | **Antibiotic discontinuation:** 1) PCT dropped by > 90% from the peak level; 2) PCT < 0.5 μg/L | Antibiotic treatment decided by the attending physician |
| Shehabi  (2014) | 1) > 18 yrs; 2) admission to ICU < 72 hrs; 3) patients receiving parenteral and/or enteral antibiotics for a suspected bacterial infection; 4) expected ICU stay > 24 hrs | **Antibiotic discontinuation:** 1) PCT < 0.10 μg/L; 2) PCT of 0.10 - 0.25 μg/L; 3) PCT dropped by > 90% compared with baseline  *Assessment of appropriateness and/or adequacy if PCT > 70% of baseline at 48 hrs | Antibiotic treatment according to the Australian Antibiotics Therapeutic Guidelines |
| Najafi  (2014) | Patients meeting at least two of the following four criterias:  1) body temperature > 380 C or < 360 C; 2) tachycardia > 90/min; 3) tachypnea > 20/min; 4) leucocytosis > 12×10^9^/L or a leftward shift > 10% band cells or leucopenia < 4×10^9^/L | 1) PCT ≤ 0.5 μg/L (Group A): antibiotics discouraged and remeasurement after 12 hrs; 2) PCT of 0.5-2 μg/L (Group B): antibiotic not discouraged and remeasurement after 8 hrs; 3) PCT ≥ 2 μg/L (Group C): antibiotic encouraged; 4) PCT remeasurement > 2 μg/L in group A and B: antibiotics encouraged; 5) PCT remeasurement < 2 μg/L in group A and B: patients undergoing close observation and PCT remeasurement until culture results obtained | Regimens according to empirical rules |
| Jong  (2016) | 1) ≥ 18 yrs; 2) antibiotic treatment < 24hrs before inclusion | **Antibiotic discontinuation:** 1) PCT dropped by 80% or more of peak value; 2) PCT of 0-5 μg/L or lower | Antibiotic treatment according to the local or national guidelines and clinical physician |
| Bloos  (2016) | Adults occuring severe sepsis or septic shock within 24 hrs before inclusion | **Antibiotic continuation:** PCT dropped by ≥ 50% compared with the baseline value on day 4;  **Antibiotic discontinuation:** 1) PCT ≤ 1 μg/L; 2) PCT dropped by ≥ 50% compared with the previous value on the other days;  *Otherwise, change or optimization of antimicrobial therapy or interventions | Antibiotic treatment decided by treating physician |

yrs, years; hrs, hours; a, 1) Procalcitonin > 1.0 ng/mL and decreased by < 10% compared the previous day; 2) A single procalcitonin measurement of > 1.0 ng/mL at baseline.
