# Supplementary figures and images for: Procalcitonin-guided antibiotic therapy in critically ill adults: a meta-analysis
Source: BMC Infect Dis. 2017 Jul 24;17:514. doi: 10.1186/s12879-017-2622-3 (PMC5525369; doi:10.1186/s12879-017-2622-3)

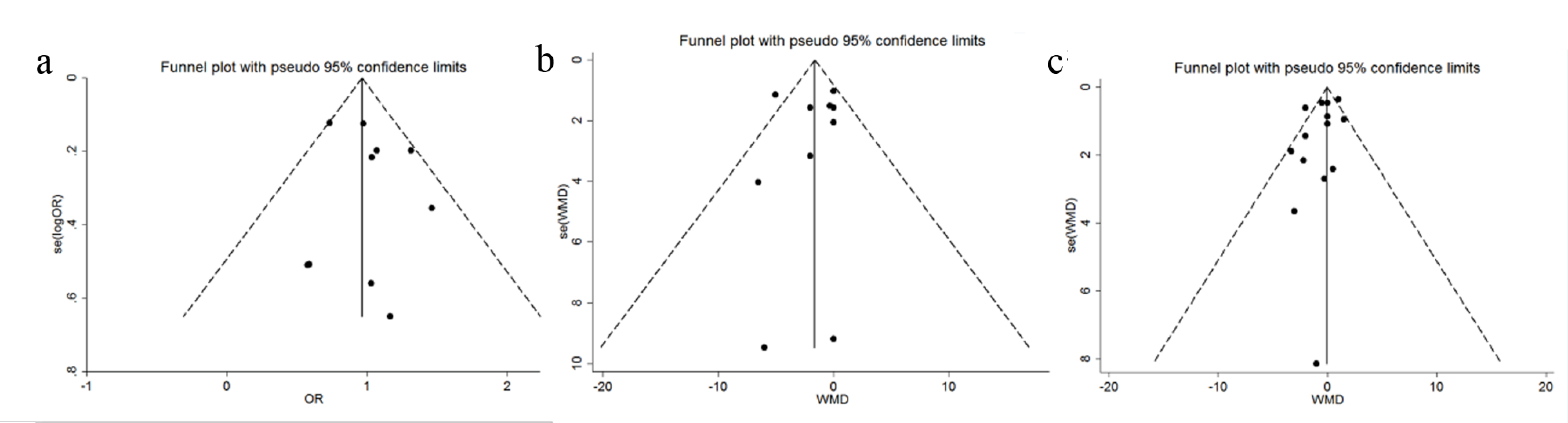

Supplement: Supplementary file 3 — Funnel plots for outcomes included more than 10 studies. (a) 28-day mortality; (b) length of hospitalization; (c) length of ICU stay. (TIFF 486 kb) [file 12879_2017_2622_MOESM3_ESM.tif]

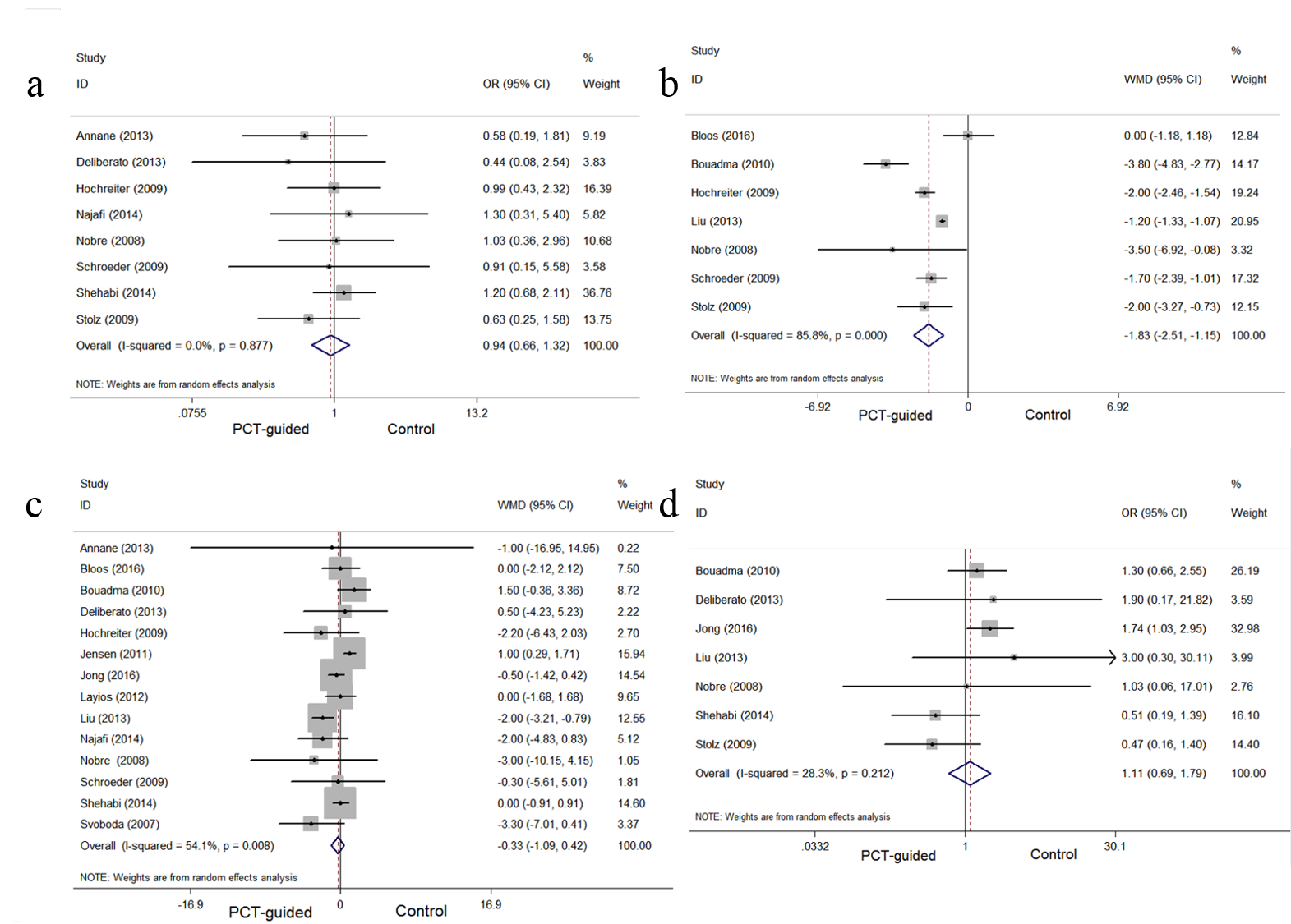

Supplement: Supplementary file 4 — Forest plots of second outcomes. (a) mortality in hospitalization; (b) total duration of antibiotic therapy; (c) length of ICU stay; (d) recurrences. (TIFF 1362 kb) [file 12879_2017_2622_MOESM4_ESM.tif]

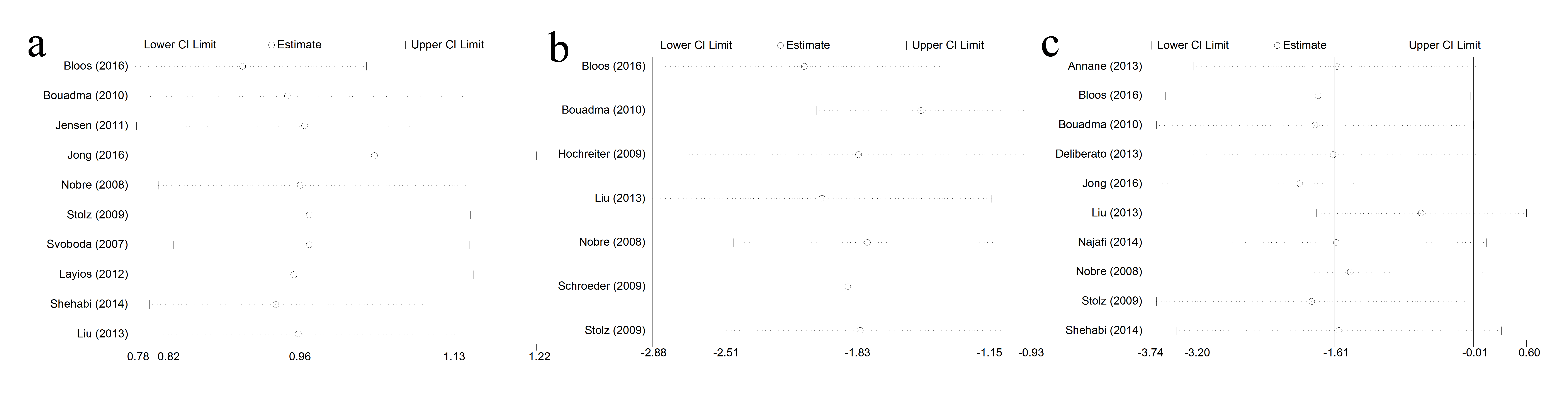

Supplement: Supplementary file 5 — Sensitivity analysis for primary outcomes by removing each trail. (a) 28-day mortality; (b) duration of antibiotic therapy for the first episode of infection; (c) length of hospitalization. (TIFF 288 kb) [file 12879_2017_2622_MOESM5_ESM.tif]

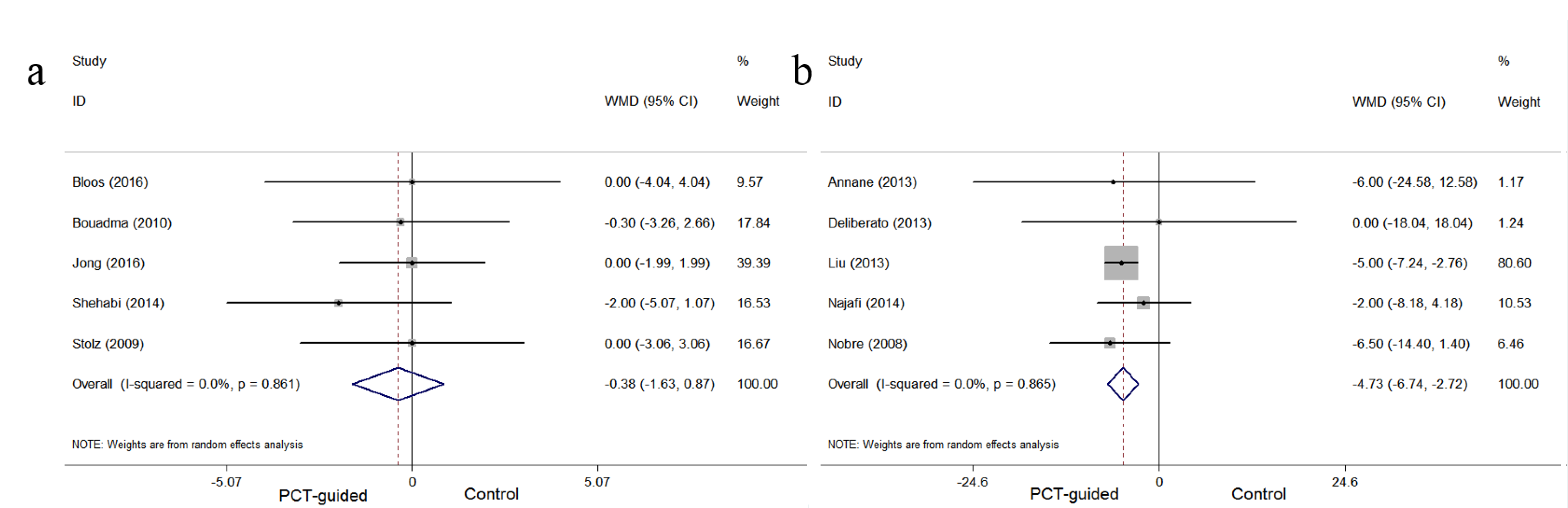

Supplement: Supplementary file 6 — Subgroup analysis for length of hospitalization: sample size >100 and sample size <100. (TIFF 511 kb) [file 12879_2017_2622_MOESM6_ESM.tif]
